# Ambient RF-EMF exposure in surgical operating rooms from telecommunication antennas and Wi-Fi sources

**DOI:** 10.3389/fpubh.2025.1721454

**Published:** 2026-01-06

**Authors:** Ramón Peyró-Sánchez, Jesus Gonzalez-Rubio, Manuel Gerónimo-Pardo, Alberto Nájera

**Affiliations:** 1 Department of Medical Sciences, Faculty of Medicine of Albacete, University of Castilla-La Mancha, Albacete, Spain; 2 Centre for Biomedical Research (CRIB), University of Castilla-La Mancha, Albacete, Spain; 3 Department of Anaesthesiology, Integrated Management Care of Albacete, Albacete, Spain

**Keywords:** ambient exposure, exposimeter, occupational exposure, operating rooms, radiofrequency electromagnetic fields (RF-EMF), surgical procedures

## Abstract

In recent years, many studies have characterized ambient exposure to radiofrequency electromagnetic fields (RF-EMF) in various environments. In hospitals, operating rooms accommodate many professionals for extended periods, often with waiting times during which mobile devices connected to wireless networks are used. These conditions could increase workers’ exposure during surgical procedures. This study aimed to characterize ambient RF-EMF exposure during surgical operations in 15 operating rooms at the General University Hospital and the Perpetuo Socorro Hospital in Albacete (Spain). Measurements were conducted using a Satimo EME Spy 140 exposimeter between 10 January and 3 March 2020. The device was configured to record data every 5 s from 08:30 to 15:00, selecting only periods corresponding to ongoing surgical operations. The exposimeter was mounted on a plastic tripod in a preferably central and unobstructed location, keeping a reasonable distance from walls and electronic equipment. In total, 67 surgical procedures were monitored, representing 120 h and 45 min of measurement. The highest recorded mean value was 58.82 μW/m^2^ on the 2.4 GHz Wi-Fi band. The exposure levels observed were comparable to those reported in other European studies conducted in indoor microenvironments. In all operating rooms analyzed, ambient exposure remained below 0.4% of the International Commission on Non-Ionizing Radiation Protection (ICNIRP, 2020) reference level, even under the least favorable conditions regarding the number of people or devices present.

## Introduction

Modern society lives in a wireless world where the spatio-temporal monitoring of ambient exposure to radio frequency electromagnetic fields (RF-EMF) is important to calm the population’s concerns about such emissions (1, 2). According to International Commission on Non-Ionizing Radiation Protection (3) and recent WHO-led systematic reviews, the only established adverse effects of RF-EMF exposure in the radiofrequency range (100 kHz–300 GHz) are thermal effects, which occur only at exposure levels far above those found in daily life or occupational environments (4). Large epidemiological and experimental reviews have not identified consistent evidence of long-term effects, including cancer, symptoms or cognitive impairment, at exposure levels below regulatory limits (5, 6). Public concern nonetheless persists, which reinforces the importance of characterizing ambient exposure in sensitive environments such as operating rooms.

Accurately estimating exposure is essential for any research into possible associations between exposure to RF-EMF and their effects on human health (7), as well as for ensuring compliance with legal or recommended exposure limits. Personal exposure studies aim to either characterize the population’s distribution of exposure (8) or measure its levels in certain environments (9). To evaluate personal exposure to these RF-EMF, they need to be suitably characterized by adopting different approaches, of which one of the most frequently used is personal exposimeters (10). These portable devices allow researchers to discriminate among different frequencies and to perform long-term recordings. However, the exposimeter measurements should be taken cautiously because of many biases and uncertainties due to mechanical errors, the design of hardware and software filters, anisotropy, the influence of bodies, etc. (11).

Many studies have been conducted to investigate potential health effects and to characterize ambient radiofrequency exposure levels in various occupational settings. According to ICNIRP (3), occupational exposure refers to situations where workers are exposed to RF-EMF due to their professional activities. Research has specifically focused on various microenvironments such as libraries, schools, public transport systems, and hospitals (12–15). Notably, a study by Massardier-Pilonchery et al. (16) emphasized occupational exposure by assessing personal RF-EMF exposure in libraries and media libraries using calibrated on-body exposimeters. This study found that occupational exposure levels were close to those of the general population, highlighting the importance of evaluating RF-EMF exposure in specific occupational settings.

At hospitals, operating rooms are places where many professionals work together in a relatively small space. During waiting periods within the surgical workflow (e.g., anesthesia stabilization or preparation phases), some staff members may briefly check their mobile devices, creating short intervals of RF-EMF activity in the operating room. Workplace risk assessments related to EMF exposure are relevant not only for operators of devices that emit EMFs, but also for support staff, maintenance personnel, and even visitors (17). In recent years, both ICNIRP and the Institute of Electrical and Electronics Engineers (IEEE) have published updated guidelines addressing worker and general population exposure (3, 18). In this context, assessing typical exposure levels in such a sensitive microenvironment as hospital operating rooms is essential to characterize the RF-EMF exposure to which healthcare workers are subjected.

However, it is important to note that the scope of this study was limited to characterizing ambient exposure to RF-EMF from common communication technologies such as antennas, mobile phones, and Wi-Fi. The specific effects of other medical devices used in operating rooms, such as electrosurgery, diathermy, and hyperthermia equipment, were not included in this study. Other studies, such as Stam and Yamaguchi-Sekino (19), have evaluated the exposure to these specific sources, providing a comprehensive understanding of the occupational RF-EMF environment in medical settings.

The main study objective was to characterize ambient exposure to RF-EMF in the operating rooms of the University Hospital Complex of Albacete (CHUA), Spain. To this end, exposure to RF-EMF was analyzed in different circumstances according to the presence of personnel, surgical operation duration, type of surgery, etc. Moreover, the obtained levels were compared to internationally recommended exposure limits.

## Methods

### Study area

This study was conducted at the CHUA, which comprises the General University Hospital (HGU) and the Perpetuo Socorro Hospital (HPS), both located in the city of Albacete (Spain). These hospitals were built in the late 20th century. The operating rooms are located within buildings featuring reinforced concrete walls, metallic-framed doors, tiled floors, and standard hospital ceilings equipped with integrated lighting and ventilation systems. These structural characteristics can influence the propagation of RF-EMF, leading to potential reflections and multipath effects. Each operating room contains a variety of electronic medical devices, although the present study primarily measured radiation originating from external antennas and internal Wi-Fi networks. While these features could affect RF-EMF propagation, they were not specifically quantified. To minimize interference, the exposimeter was strategically placed on a plastic tripod at a central point within each operating room, away from electronic devices, metal objects, and walls.

The study initially included 15 operating rooms: ten at the HGU (Q0–Q9) on the first floor and five at the HPS (Q21–Q25) on the second floor. At the HGU, Q0 was used for gynecology surgery, Q1 for pediatric surgery and neurosurgery, Q2 for orthopedics and chest surgery, Q3 for orthopedic surgery, Q4 for emergency surgery, Q5 for vascular surgery, Q6 for otorhinolaryngology and maxillofacial surgery, Q7 for general surgery, Q8 for urological surgery, and Q9 for obstetrics. At the HPS, Q21 was used for otorhinolaryngology, Q22 for general surgery, Q23 for traumatology, Q24 for urology, and Q25 for ophthalmology.

**Figure 1:**
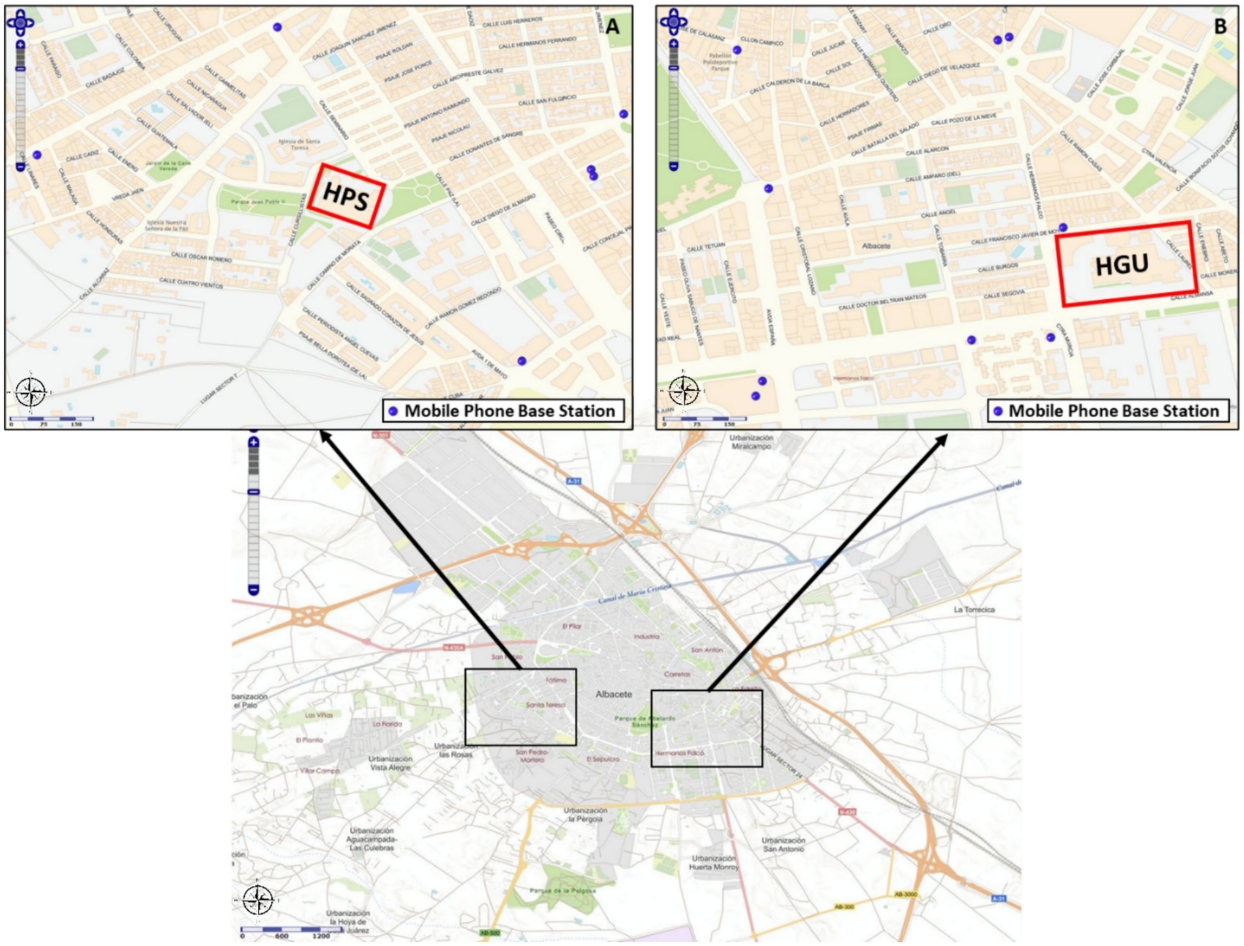
Location of the HPS **(A)** and the HGU **(B)** in the city of Albacete, and the nearest mobile phone antennae. Maps used with permission from Infoanten as – Geoportal MINETUR, Gobierno de España (https://geoportal.minetur.gob.es/VCTEL/vcne.do).

At the HPS, all operating rooms face south, whereas at the HGU, Q0–Q6 face north and Q7–Q8 face east. Mobile communication antennas are located near both hospitals. The HPS is surrounded by several antennas except to the south, the direction in which its operating rooms face. In contrast, near the HGU, only two nearby antennas are located to the north and southeast (Figure 1).

Finally, due to scheduling and operational constraints, measurements were carried out in 10 operating rooms: seven at the HGU (Q1, Q2, Q4, Q5, Q6, Q7, and Q8) and three at the HPS (Q21, Q22, and Q23).

The results obtained according to operating room are shown in Tables 1 and 2. Of all the surgical operations, eight took place in Q1, seven in Q2, five in Q4, two in Q5, one in Q6, five in Q7, two in Q8, four in Q21, 12 in Q22 and three in Q23. No valid data were recorded or surgical operations did not take place in operating rooms Q0, Q3, Q9, Q24 and Q25.

**Table 3:** Descriptive statistics according to operating room number (HGU).

		Q1	Q2	Q4	Q5	Q6	Q7	Q8
GSM + UMTS 900 (UL)	Mean	1.5	2.4	2.5	1.4	19.3	1.2	0.9
	Median	0.3	0.3	0.2	0.3	0.8	0.3	0.2
	95%CI	1.1–2.0	1.1–3.6	1.4–3.6	1.1–1.6	1.4–2.5	0.8–1.5	0.6–1.2
	P95	4.7	3.5	4.0	6.1	44.1	2.9	2.2
GSM + UMTS 900 (DL)	Mean	13.8	18.4	4.5	1.6	0.1	0.8	0.4
	Median	11.6	17.4	4.5	1.5	0.1	0.6	0.4
	95%CI	13.7–13.9	18.3–18.6	4.5–4.5	1.6–1.6	0.1–0.2	0.8–0.8	0.4–0.4
	P95	28.7	29.8	6.6	2.2	0.4	1.8	0.6
GSM 1800 (UL)	Mean	3.7	6.2	14.9	37.4	4.5	16.1	18.3
	Median	0.5	0.4	0.6	0.5	0.5	0.3	0.3
	95%CI	3.2–4.1	4.5–8.0	12.2–17.5	33.5–41.2	3.4–5.6	13.1–19.0	12.6–24.0
	P95	9.0	16.3	61.3	191.7	18.7	59.7	62.8
GSM 1800 (DL)	Mean	14.6	13.4	3.4	1.4	0.4	0.5	0.5
	Median	13.8	13.4	3.2	1.3	1.7	0.5	0.4
	95%CI	14.5–14.7	13.2–13.6	3.4–3.5	1.4–1.4	0.1–0.8	0.5–0.6	0.5–0.5
	P95	28.7	21.0	5.9	1.9	3.1	0.9	0.8
UMTS 2100 (UL)	Mean	2.4	0.6	2.5	5.0	50.5	5.0	6.6
	Median	0.3	0.2	0.4	0.4	0.7	0.8	0.7
	95CI%	1.7–3.1	0.4–0.8	1.3–3.7	1.8–8.2	28.5–72.4	3.9–6.0	4.2–6.0
	P95	8.3	3.2	6.0	20.7	192.5	20.5	30.1
UMTS 2100 (DL)	Mean	1.6	4.6	0.3	2.3	-	0.1	0.1
	Median	1.1	3.8	0.3	0.2	-	0.1	0.1
	95%CI	1.5–1.6	4.5–4.7	0.3–0.4	2.2–2.3	-	0.1–0.1	0.1–0.1
	P95	4.0	9.2	0.5	0.4	-	0.2	0.1
2.4 GHz Wi-Fi	Mean	41.8	12.8	10.8	27.8	29.4	8.7	160.7
	Median	2.2	1.4	2.2	6.6	0.6	0.5	1.1
	95%CI	39.8–43.8	6.3–19.3	8.6–12.6	24.3–31.3	20.7–37.9	7.1–10.2	141.1–180.3
	P95	261.5	16.6	27.6	96.0	73.7	22.0	1173.5

Power density in μW/m^2^.

### Exposimeter

To determine personal exposure, an exposimeter EME Spy 140 (Satimo) was used, which records 14 frequency bands (from 88 MHz to 5 GHz) with a maximum sensitivity of 0.005 V/m (corresponds to a detection limit of approximately 0.0665 μW/m^2^ for power density). Its dimensions are 168.5 × 79 × 46.7 mm and it weighs 400 g. It is able to take up to 60,000 measurements within intervals varying from 4 to 255 s.

Of these 14 frequency bands, the 2G Wi-Fi, GSM and UTMS UL (uplink) and DL (downlink) bands (900, 1800 and 2,100 Hz) were taken into account.

**Figure 2:**
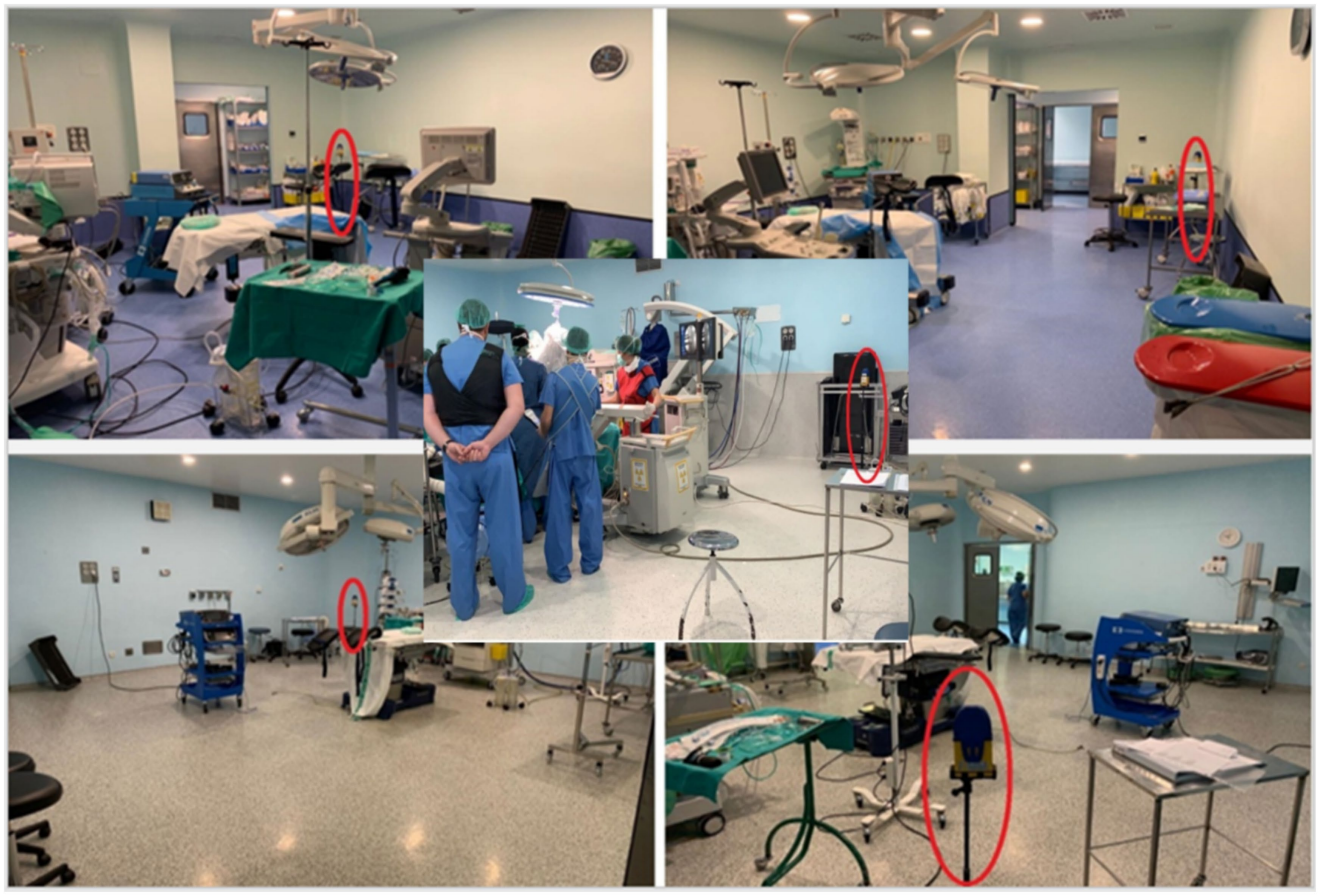
Location of the personal exposimeter on a plastic tripod in different operating rooms and situations.

To limit possible interferences while taking measurements, the exposimeter was placed on a plastic tripod, positioned as far away as possible from walls and other electrical devices to avoid interferences (Figure 2).

The exposimeters used in our study were previously calibrated by the French company Antenessa/Satimo.

### Measurements and data processing

The exposimeter was configured to record data every 5 s between 08:30 and 15:00, from 10 January to 3 March 2020. It was mounted on a plastic tripod in a preferably central and unobstructed location, depending on surgical needs, while maintaining a minimum distance of ≥30 cm from walls and ≥1 m from electronic equipment (10). Measuring ambient exposure at a fixed point was chosen instead of body-worn measurement to avoid interference or shadowing effects from the body or other equipment.

Two surgical operations were generally performed each day. Although the exposimeter was programmed to measure throughout the morning period, only the intervals corresponding to active surgical procedures were considered for analysis. The duration of each operation was defined from the time the patient entered the operating room to the time they left it. Measurements recorded between operations or during procedures in which data collection was interrupted or failed were excluded from the analysis.

To characterize the different conditions, the following variables were considered:

- Hospital: operations performed at either the HGU or the HPS.
- Operating room: as previously described, Q0–Q6 at the HGU face north and are located further from the nearest antennas, while Q7 and Q8 face east. All HPS operating rooms face south.
- Number of people: operations with more or fewer than 10 people present.
- Mean duration: operations classified as *short* or *long* depending on whether they lasted less or more than 2.5 h.
- Type of operation: programmed and uncomplicated operations versus those classified as urgent or with complications.

Mobile phones are not used during active surgical activity. However, during predefined waiting periods (e.g., anesthesia stabilization or instrument preparation), staff may briefly check their devices, mainly for messaging or coordination purposes using Wi-Fi/data connection.

Despite the high sensitivity of the exposimeter (0.005 V/m), a substantial proportion of measurements in several frequency bands were below the device’s detection threshold. These values were recorded and assigned a nominal “non-detect” (ND) category for further analysis. Different methodologies exist for handling non-detects (20), depending on their proportion within the dataset.

The Kolmogorov–Smirnov test was used to assess data normality, which indicated that the variables did not follow a normal distribution. Consequently, non-parametric tests were applied: the Mann–Whitney *U* test and the Kruskal–Wallis test, as appropriate. A significance level of *p* < 0.05 was adopted. All statistical analyses were conducted using SPSS version 24.0 (SPSS Inc., Chicago, IL, USA) and R version 4.1.2 (R Foundation for Statistical Computing, Vienna, Austria).

## Results

Twenty-six days and 67 surgical operations were recorded, totaling 120 h and 45 min. Forty-two operations took place at the HGU and 25 at the HPS. Of all the surgical operations, 49 lasted less than 2.5 h and 18 took longer. There were fewer and more than 10 people in operating rooms during 55 and 12 surgical operations, respectively. Only one operation was urgent; the rest were scheduled and presented no complications. Finally, 49 surgical operations (96 h and 18 min) were validated, and the rest were ruled out due to errors in registrations (data recording process encountered issues such as incorrect timestamps), movements in the exposimeter, operation requirements, failed measurements (where the exposimeter did not record any data due to device malfunctions or other technical problems), etc., with 30 at the HGU and 19 at the HPS.

Tables 3, 4 show the percentage of valid and ND values for each frequency band. A high proportion of ND values in uplink bands (UL) reflects that staff were generally not using their mobile devices during surgical procedures. Because the UL 900 MHz and UL 2100 MHz bands showed 81.3 and 94.0% ND values, respectively, and following a < 35% threshold for inclusion, these bands were removed from the analysis to avoid bias.

**Table 4:** The total “non-detect (ND)” percentages when the study ended.

Band	Frequency (MHz)	% Non-detects (ND)
GSM + UMTS 900 (UL)	880–915	81.3
GSM + UMTS 900 (DL)	925–960	4.0
GMS 1800 (UL)	1710–1785	27.0
GMS 1800 (DL)	1805–1880	4.3
UMTS 2100 (UL)	1920–1980	94.0
UMTS 2100 (DL)	2,110–2,170	17.7
2.4 GHz Wi-Fi	2,400–2,500	12.5

**Table 5:** Non-detects (ND) according to hospital.

	GSM + UMTS 900 (UL)	GSM + UMTS 900 (DL)	GSM 1800 (UL)	GSM 1800 (DL)	UMTS 2100 (UL)	UMTS 2100 (DL)	2.4 GHz Wi-Fi
HGU	76.4	5.5	17.6	6.0	92.2	15.9	7.4
HPS	93.8	0.0	50.9	0.0	99.2	4.5	25.7

### Hospital

Table 5 shows the descriptive statistics for each hospital where the sample was taken. The highest mean value was 48.4 μW/m^2^ for the 2.4 GHz Wi-Fi band at the HPS with a P95 of 90.8 μW/m^2^ and the 95% confidence interval (95%CI) was between 40.7 μW/m^2^ and 56.1 μW/m^2^. The lowest mean value was 0.8 μW/m^2^ for the UMTS 2100 (DL) band at the HPS with a P95 of 2.7 μW/m^2^ and the 95%CI between 0.7 μW/m^2^ and 0.8 μW/m^2^.

**Table 6:** The descriptive statistics according to hospital.

	Mean		95%CI		Median		IQR		P95	
	HGU	HPS	HGU	HPS	HGU	HPS	HGU	HPS	HGU	HPS
GSM + UMTS 900 (UL)	5.3	37.3	4.1–6.6	28.5–46.2	0.3	1.2	0.8	11.7	8.9	151.4
GSM + UMTS 900 (DL)	8.0	6.1	7.9–8.1	6.0–6.2	5.1	3.1	12.1	4.0	25.1	22.0
GSM 1800 (UL)	10.9	22.7	10.1–11.7	19.8–25.6	0.4	0.8	1.3	4.9	42.1	78.5
GSM 1800 (DL)	7.7	3.5	7.6–7.8	3.5–3.5	4.2	2.2	12.8	2.1	23.9	10.5
UMTS 2100 (UL)	10.8	2.6	7.5–14.1	0.2–5.4	0.5	0.1	1.9	0.2	25.1	2.1
UMTS 2100 (DL)	1.3	0.8	1.2–1.3	0.7–0.8	0.5	0.4	1.5	0.7	4.7	2.7
2.4 GHz Wi-Fi	38.1	48.4	35.9–40.3	40.7–56.1	1.7	2.2	5.7	7.5	133.1	90.8

Power density in μW/m^2^.

The possible differences in frequency bands were studied according to the hospital where the surgical operation was performed. Significant differences were found for all the frequency bands (*p* < 0.05).

### Number of people in operating rooms

The results for the number of people in operating rooms during surgical procedures are shown in Table 6. The highest mean value was 43.5 μW/m^2^ for the 2.4 GHz Wi-Fi band during surgeries with fewer than 10 people, with a P95 of 101.9 μW/m^2^ and a 95% CI of 40.0–46.9 μW/m^2^. Conversely, the lowest mean value was 0.6 μW/m^2^ for the UMTS 2100 (DL) band during surgeries with 10 or more people, with a P95 of 2.7 μW/m^2^ and a 95% CI of 0.55–0.57 μW/m^2^. For each frequency band, mean RF-EMF exposure during surgeries with fewer than 10 people was compared with surgeries involving 10 or more people. Significant differences were observed in all frequency bands except GSM 1800 UL (*p* > 0.05). When more than 10 people were present, a group of students was often included, reducing the likelihood of personal mobile device use during teaching phases.

**Table 7:** Descriptive statistics according to number of people (*n* < 10 and *n* > 10).

	Mean		95%CI		Median		P95	
	<10	>10	<10	>10	<10	>10	<10	>10
GSM + UMTS 900 (UL)	7.5	9.3	5.9–9.1	6.9–11.7	0.3	0.4	14.5	16.6
GSM + UMTS 900 (DL)	8.3	5.3	8.2–8.3	5.2–5.3	3.6	4.7	26.0	13.8
GSM 1800 (UL)	14.9	9.5	13.8–15.9	8.2–10.9	0.5	0.4	5.8	3.3
GSM 1800 (DL)	6.3	7.0	6.2–6.3	6.9–7.1	2.5	4.5	22.5	21.4
UMTS 2100 (UL)	3.7	16.0	3.0–4.4	10.2–21.6	0.5	0.4	15.7	39.5
UMTS 2100 (DL)	1.3	0.6	1.3–1.3	0.6–0.6	0.4	0.4	4.7	1.4
2.4 GHz Wi-Fi	43.5	34.3	40.0–46.9	31.9–36.8	1.9	1.4	101.9	156.6

Power density in μW/m^2^.

### Surgical operation duration

Table 7 provides the results for this variable. Of all the surgical operations, 39 lasted less than 2.5 h (150 min), but 10 lasted longer and were all performed at the HGU. The highest mean value was 48.9 μW/m^2^ for the 2.4 GHz Wi-Fi band during the surgical operations lasting less than 150 min, with a P95 of 98.8 μW/m^2^ and a 95%CI between 44.3 μW/m^2^ and 53.5 μW/m^2^. The lowest mean value was 1.1 μW/m^2^ for the UMTS 2100 (DL) band during the operations lasting more than 150 min, with a P95 of 3.7 μW/m^2^ and a 95%CI between 1.04 μW/m^2^ and 1.07 μW/m^2^.

**Table 1:** Descriptive statistics according to surgical operation duration (<150 and >150 min).

	Mean		95%CI		Median		P95	
	<150	>150	<150	>150	<150	>150	<150	>150
GSM + UMTS 900 (UL)	11.0	6.8	8.6–13.4	5.1–8.6	0.3	0.3	27.6	12.3
GSM + UMTS 900 (DL)	7.0	7.9	6.9–7.1	7.8–8.0	3.8	4.2	22.5	26.0
GSM 1800 (UL)	15.2	11.2	13.8–16.6	10.3–12.1	0.4	0.5	55.0	42.1
GSM 1800 (DL)	5.0	8.2	4.9–5.0	8.1–8.3	2.5	4.0	16.1	25.5
UMTS 2100 (UL)	3.4	13.5	2.3–4.5	9.0–18.1	0.4	0.5	8.6	31.5
UMTS 2100 (DL)	1.1	1.1	1.1–1.1	1.0–1.1	0.4	0.5	4.7	3.7
2.4 GHz Wi-Fi	48.9	32.2	44.3–53.5	30.4–34.1	1.9	1.5	98.9	141.5

Power density in μW/m^2^.

No significant differences were found for frequency bands UMTS 2100 (DL) and 2.4 GHz Wi-Fi (*p* > 0.05). For the other bands, significant differences in exposure appeared depending on whether the operation lasted more or less than 2.5 h (*p* < 0.05).

### Operating rooms

The results obtained according to operating room appear in Tables 1, 2. Of all the surgical operations, eight took place in Q1, seven in Q2, five in Q4, two in Q5, one in Q6, five in Q7, two in Q8, four in Q21, 12 in Q22 and three in Q23. No valid data were recorded or surgical operations did not take place in operating rooms Q0, Q3, Q9, Q24 or Q25. The highest mean value was 160.7 μW/m^2^ for the 2.4 GHz Wi-Fi band during the surgical operations that took place in Q8, with a P95 of 1173.5 μW/m^2^ and a 95%CI between 141.1 μW/m^2^ and 180.3 μW/m^2^. The lowest mean value was 0.1 μW/m^2^ for the UMTS 2100 (DL) band during the surgical operations that took place in Q8, with a P95 of 0.1 μW/m^2^ and a 95%CI between 0.105 μW/m^2^ and 0.107 μW/m^2^.

This was an interesting variable for seeking differences between operating rooms Q7 and Q8 and the others because the orientation of Q7 and Q8 differs. Differences were sought between exposure to the different RF-EMF bands in all the operating rooms and per hospital. Significant differences appeared for all the bands among all the operating rooms regardless of being located at the HPS or the HGU, and Q7 and Q8 were at the HGU (*p* < 0.05) (Table 7).

**Table 2:** Descriptive statistics according to operating room number (HPS).

		Q21	Q22	Q23
GSM + UMTS 900 (UL)	Mean	13.6	52.3	1.1
	Median	0.8	3.4	0.3
	95%CI	9.0–18.2	39.2–65.3	0.7–1.5
	P95	94.8	255.4	4.4
GSM + UMTS 900 (DL)	Mean	16.9	2.8	3.1
	Median	15.3	2.4	2.9
	95%CI	16.7–17.1	2.6–2.8	3.0–3.1
	P95	29.8	5.4	4.9
GSM 1800 (UL)	Mean	10.6	32.6	18.5
	Median	0.3	1.7	1.8
	95%CI	8.0–13.1	27.3–38.0	14.8–22.2
	P95	33.3	108.2	77.5
GSM 1800 (DL)	Mean	8.5	1.8	2.5
	Median	8.6	1.8	2.5
	95CI%	8.4–8.6	1.8–1.8	2.5–2.6
	P95	12.6	2.9	4.0
UMTS 2100 (UL)	Mean	0.1	6.3	0.4
	Median	0.1	0.4	0.2
	95%CI	0.1–0.2	0.8–13.3	0.2–1.0
	P95	0.2	35.7	-
UMTS 2100 (DL)	Mean	2.0	0.4	0.3
	Median	1.7	0.3	0.3
	95%CI	2.0–2.1	0.4–0.4	0.3–0.3
	P95	4.5	1.0	0.9
2.4 GHz Wi-Fi	Mean	40.6	58.8	16.4
	Median	2.5	2.5	1.1
	95%CI	19.3–60.8	49.1–68.6	12.6–20.1
	P95	52.4	134.3	64.2

Power density in μW/m^2^.

## Discussion

This study aimed to characterize ambient exposure to RF-EMF in the operating rooms of the CHUA. Exposure levels were analyzed under different conditions according to hospital, operating room, number of people present, duration, and type of surgical procedure, and were compared with international reference limits. The research focused on ambient exposure during surgical operations, a particularly relevant scenario where many healthcare professionals coincide and occasionally use mobile devices connected to wireless networks during waiting periods. These circumstances could potentially influence exposure conditions in operating rooms.

Ambient RF-EMF levels remained low despite the presence of multiple wireless sources. This can be explained by the low transmission power and duty cycle of hospital Wi-Fi systems, the attenuation caused by shielding materials and structural barriers of operating rooms, and the fact that UL activity only increases during short, intermittent periods when mobile devices are briefly used. These conditions inherently limit continuous exposure and help explain the variability observed between frequency bands.

Although the measurements primarily reflected ambient RF-EMF levels generated by external antennas and internal Wi-Fi networks, sources that are not expected to pose additional risks to individuals with medical implants beyond those faced by the general population, it is important to recognize that healthcare workers with active medical devices may have specific safety concerns. This consideration is related to the susceptibility of certain active medical implants (e.g., pacemakers, neurostimulators or infusion pumps) to electromagnetic interference, as described in device safety standards and ICNIRP guidance, even though the exposure levels measured in our study were far below regulatory limits. This aspect was beyond the scope of the present study and should be explored in future research.

Measuring exposure at a fixed central point was necessary to avoid the practical difficulties and potential errors associated with body-worn measurements in such a controlled environment. The EME Spy 140 exposimeter was selected because of its compact design, ease of use, and high sensitivity, which make it particularly suitable for complex environments such as operating rooms where sterility, accessibility, and safety standards must be maintained. Positioning the exposimeter on a tripod at a central point allowed for consistent and reliable characterization of ambient exposure (21).

Even under the least favorable conditions, ambient exposure levels remained extraordinarily below the ICNIRP (3) occupational reference limits, confirming the absence of any health risk. According to ICNIRP (3), the reference levels for occupational exposure to radiofrequency electromagnetic fields range from 22.5 W/m^2^ at 900 MHz to 50 W/m^2^ for frequencies above 2 GHz, including the 2.4 GHz Wi-Fi band. These results are consistent with previous studies conducted in various indoor microenvironments across Europe (22).

Exposure values varied considerably among frequency bands. The maximum instantaneous value recorded was 31188.3 μW/m^2^ for the 2.4 GHz Wi-Fi band, while the minimum detected value was 0.1 μW/m^2^ in several bands. The consistently higher levels observed in the 2.4 GHz Wi-Fi band likely reflect the predominant use of Wi-Fi for data exchange in hospital environments, where multiple devices (e.g., smartphones, laptops, tablets, and medical workstations) remain continuously connected to access clinical information, synchronize data, or receive notifications. The highest mean value was 48.4 μW/m^2^ for the 2.4 GHz band at the HPS and 34.1 μW/m^2^ at the HGU, while the lowest mean value was 0.1 μW/m^2^ for the UMTS 2100 (DL) band. These results highlight the importance of evaluating multiple frequency bands to fully understand RF-EMF dynamics in hospital environments.

When comparing our results with previous research, we found good agreement with studies by Sagar et al. (9, 15) and Ramírez-Vázquez et al. (23). These studies demonstrated that exposure levels to RF-EMF vary widely depending on the environment. For instance, Sagar et al. (15) reported average exposure levels between 0.23 V/m and 1.85 V/m in outdoor microenvironments, where mobile phone base stations were the predominant sources of exposure. Similarly, Sagar et al. (9) observed mean exposure levels of 0.29 V/m (223.1 μW/m^2^) in indoor and 0.54 V/m (773.5 μW/m^2^) in outdoor environments across Europe, both well below the established limits. Ramírez-Vázquez et al. (23) also concluded that exposure levels in most settings remain substantially below ICNIRP reference values.

In our study, the maximum recorded value of 31188.3 μW/m^2^ (2.4 GHz Wi-Fi band, Q22) was well below 1% of the ICNIRP reference level. Exposure to RF-EMF was highly variable across hospitals, frequency bands, and operational conditions such as duration, occupancy, and operating room.

When analyzed by hospital, significant differences were observed among frequency bands. The highest mean value (48.4 μW/m^2^) was again found for the 2.4 GHz Wi-Fi band at the HPS, while the lowest mean value (0.8 μW/m^2^) corresponded to the UMTS 2100 (DL) band. This suggests that structural and spatial differences between hospital buildings can influence the distribution of RF-EMF exposure.

Significant differences (*p* < 0.05) were observed depending on whether fewer or more than 10 people were present in the operating room. RF-EMF exposure tended to be higher during procedures with fewer than 10 people, particularly in the 2.4 GHz Wi-Fi band, whereas lower values were recorded during surgeries with more than 10 people for the UMTS 2100 (DL) band. Short intermittent pauses inherent to surgical workflow (e.g., preparation, equipment changes, documentation) are the periods in which personal mobile device use is most likely to occur, explaining transient UL increases. The duration of these pauses varies substantially across procedures and depends on surgical complexity and coordination among staff. Quantifying waiting time as a percentage of total surgical duration was beyond the scope of the study, as this was not systematically recorded. Additionally, when more than 10 people were present, groups of students were often included, reducing personal device use during active teaching phases, which may contribute to lower RF-EMF levels.

The higher exposure observed during procedures with fewer than 10 staff members suggests increased likelihood of personal mobile device use during waiting periods. In contrast, procedures with more than 10 people often involved teaching activities and the presence of students, during which mobile device use is discouraged, reducing UL activity. Only the GSM 1800 UL band showed no significant differences, likely because GSM voice calls were rare, whereas the highest values were consistently recorded in the 2.4 GHz Wi-Fi band, reflecting the prevalence of Wi-Fi–based data communication in current hospital networks.

The duration of surgical operations also affected ambient RF-EMF levels. Significant differences (*p* < 0.05) were observed for most frequency bands depending on whether operations lasted more or less than 2.5 h. The 2.4 GHz Wi-Fi band again showed the highest mean values during shorter procedures (<150 min), which may relate to the intermittent use of wireless devices during brief waiting periods. These findings underscore the need to consider operation duration when evaluating exposure patterns in medical environments. The thresholds chosen for occupancy (10 people) and duration (2.5 h/150 min) were based on empirical observations during data collection and should be refined in future research.

When exposure levels were compared across operating rooms, significant differences were identified (*p* < 0.05). Operating room Q8 showed the highest mean value for the 2.4 GHz Wi-Fi band, while the lowest mean corresponded to the UMTS 2100 (DL) band. Orientation and structural variability also played a role. Q7 and Q8, which face east unlike most others, displayed distinctive exposure levels. These results suggest that the spatial layout, equipment distribution, and architectural characteristics of each room can significantly affect RF-EMF propagation. Therefore, spatial and structural variability must be taken into account when assessing ambient exposure in complex indoor settings.

Differences among operating rooms, even within the same hospital, are likely due to spatial factors such as room geometry, shielding materials, and the location of Wi-Fi access points. The behavior of the different frequency bands also varies depending on their propagation properties: lower-frequency cellular bands penetrate structural barriers more effectively, while 2.4 GHz Wi-Fi is strongly influenced by the distance to the nearest access point and signal attenuation caused by metal structures and medical equipment.

Ambient RF-EMF variability across operating rooms may also be influenced by architectural or logistical factors that were not directly assessed in this study, such as the location of Wi-Fi access points or shielding from structural elements. The fixed-point measurements used here reflect ambient environmental exposure rather than maximum personal exposure of staff members. Future studies could complement this environmental approach with total exposure metrics (sum of all frequency bands) and wearable measurements to allow direct comparison with personal exposure studies. The fact that significant differences were not always consistent across frequency bands likely reflects different transmission behaviors (e.g., UL vs. DL activity and Wi-Fi duty cycles) rather than inconsistent measurement performance.

The use of personal exposimeters presents several advantages, including their small size, ease of use, and ability to record large datasets in diverse conditions (24). However, this study also faced several limitations. Data collection spanned 26 days and included 67 surgical operations (120 h and 45 min). Of these, 49 operations (96 h and 18 min) were ultimately analyzed due to the following issues:

- Hardware failures: the device occasionally stopped recording, preventing data collection during some operations.
- Software errors: on certain days, data could not be transferred, resulting in the loss of complete daily records.
- Incomplete recordings: in some cases, the exposimeter was activated after surgery had begun, leading to partial datasets that were excluded from analysis.

Only one urgent operation (defenestration in Q4, HGU) was recorded, involving more than 10 participants and lasting over 150 min. This single sample was excluded from comparative analyses due to its limited representativeness. Increasing the number of monitored days could address this limitation in future studies.

Like all exposimeter-based research, our measurements were subject to potential sources of bias, including mechanical errors, anisotropy, and hardware or software malfunction (12, 25, 26). The influence of the body and directional bias were mitigated by fixing the exposimeter at a constant height on a tripod rather than using body-worn measurements. However, mechanical disturbances occasionally occurred when the tripod was unintentionally moved, which may have introduced minor variability in recorded values.

Although the sample size was relatively large, a high percentage of ND values was found in some UL bands, exceeding 90% for UMTS 2100. Various statistical methods exist to handle values below the detection threshold (20). In this study, data were excluded from analysis when ND values represented more than 35% of the total to avoid bias.

Additionally, differences in building materials or the orientation of mobile phone antennas relative to each hospital were not explicitly analyzed and could partly explain some inter-hospital variability. Moreover, while the number of people in the operating rooms often reflected staff presence, it was occasionally influenced by teaching activities involving students.

One additional limitation is the absence of measurements for the 5 GHz Wi-Fi band. This frequency band was not assessed because, during the experimental phase of data collection, it had not yet been deployed or was not in active use in the operating rooms studied. The hospital wireless infrastructure at that time operated primarily in the 2.4 GHz band. However, conducting measurements in a pre-5G deployment scenario provides a valuable baseline for future comparison. Follow-up studies should consider including higher-frequency Wi-Fi bands, such as 5 GHz, as well as emerging 5G technologies, to provide a more comprehensive assessment of RF-EMF exposure in clinical environments.

In conclusion, this study provides a detailed evaluation of ambient RF-EMF exposure during surgical operations in hospital operating rooms. Although the highest exposure levels were associated with the 2.4 GHz Wi-Fi band, all measured values were well below the limits established by ICNIRP (3). These findings align with previous research conducted in similar environments and reaffirm that ambient RF-EMF exposure in hospitals poses no identifiable health risk to healthcare workers or patients. Continued monitoring and characterization of RF-EMF exposure in healthcare settings remain essential to ensure the long-term safety and well-being of medical personnel and patients.

The scientific evidence provided by this study may also serve as a foundation for developing occupational health communication strategies aimed at hospital staff and patients. Presenting clear, data-driven information that confirms RF-EMF exposure levels in operating rooms are well below international safety limits could help address concerns, promote informed awareness, and reinforce confidence in the safety of healthcare environments, particularly among individuals with medical implants or heightened sensitivity to electromagnetic exposure.

## Data Availability

The raw data supporting the conclusions of this article will be made available by the authors, without undue reservation.
